# Adenoviral Vectors Meet Gene Editing: A Rising Partnership for the Genomic Engineering of Human Stem Cells and Their Progeny

**DOI:** 10.3390/cells9040953

**Published:** 2020-04-13

**Authors:** Francesca Tasca, Qian Wang, Manuel A.F.V. Gonçalves

**Affiliations:** Department of Cell and Chemical Biology, Leiden University Medical Center, Einthovenweg 20, 2333 ZC Leiden, The Netherlands

**Keywords:** genome editing, stem cells, induced pluripotent stem cells, programmable nucleases, CRISPR nucleases, adenoviral vectors, high-capacity adenoviral vectors, nickases, base editors, prime editors

## Abstract

Gene editing permits changing specific DNA sequences within the vast genomes of human cells. Stem cells are particularly attractive targets for gene editing interventions as their self-renewal and differentiation capabilities consent studying cellular differentiation processes, screening small-molecule drugs, modeling human disorders, and testing regenerative medicines. To integrate gene editing and stem cell technologies, there is a critical need for achieving efficient delivery of the necessary molecular tools in the form of programmable DNA-targeting enzymes and/or exogenous nucleic acid templates. Moreover, the impact that the delivery agents themselves have on the performance and precision of gene editing procedures is yet another critical parameter to consider. Viral vectors consisting of recombinant replication-defective viruses are under intense investigation for bringing about efficient gene-editing tool delivery and precise gene-editing in human cells. In this review, we focus on the growing role that adenoviral vectors are playing in the targeted genetic manipulation of human stem cells, progenitor cells, and their differentiated progenies in the context of in vitro and ex vivo protocols. As preamble, we provide an overview on the main gene editing principles and adenoviral vector platforms and end by discussing the possibilities ahead resulting from leveraging adenoviral vector, gene editing, and stem cell technologies.

## 1. Introduction

### 1.1. The Main Gene Editing Principles Based on Programmable Nucleases and Their Key Pros and Cons

Commonly, gene editing is triggered after programmable nucleolytic enzymes bind to predefined chromosomal sequences and locally generate double-stranded or single-stranded DNA breaks (DSBs or SSBs, respectively). The ensuing mending of these chromosomal breaks by cellular DNA repair mechanisms leads to the installation of targeted genomic changes whose extent can span from single to thousands of base pairs (bps).

Gene editing endeavors can disable a coding sequence (knockout) or remove specific genomic tracts. Moreover, they can equally restore a coding sequence or insert into specific genomic locations new genetic information (knock-in) present in exogenous (donor) DNA molecules. Typically, DNA editing strategies that knock-out or restore endogenous coding sequences involve the transfer of programmable nucleases that generate frameshifting insertions and deletions (indels) after the repair of targeted DSBs by non-homologous end joining (NHEJ) pathways. These include, classic NHEJ (cNHEJ) and alternative NHEJ (alt-NHEJ) pathways such as microhomology-mediated end-joining (MMEJ) and single-strand annealing (SSA) [1]. The cNHEJ is the most active and fast-acting of the DNA repair pathways in mammalian cells often resulting in no or limited end-processing by exonucleases prior to ligation of chromosomal ends [1]. Importantly, chromosomal ligation products containing indels can be generated [1], especially in the presence of a programmable nuclease that re-cleaves precisely ligated products until an indel disrupts its target site and becomes “fixed” in the cell population. It is also noteworthy mentioning that; (i) the target site sequences, (ii) the class of programmable nuclease employed, and (iii) the type of repair mechanism engaged in DSB repair, all contribute to different indel profiles which vary considerably in length and nucleotide composition [1,2]. Yet, depending to some extent on microhomologies, the targeting of specific sequences by a programmable nuclease can yield specific indels in a high frequency of modified alleles [3,4,5,6,7].

Indels resulting from NHEJ-mediated repair of targeted DSBs can be exploited for disrupting non-coding elements (e.g., splicing motifs to induce exon-skipping) or reframing coding sequences that rescue endogenous gene expression via bypassing preexisting nonsense mutations (i.e., premature stop codons) [8,9]. Alternatively, indels can be exploited for disrupting coding sequences that knockout endogenous gene expression via installing stop codons that induce nonsense-mediated mRNA decay (NMD) [9,10,11]. However, it is important to mention recent research demonstrating the existence of an evolutionary conserved NMD-dependent mechanism in which the presence of a nonsense mutation in a gene can activate transcription of related genes whose products functionally complement the mutant gene [12,13]. Another cautionary note concerns other recent findings in which DSB-derived indels in coding sequences can generate transcripts yielding various types of aberrant gene products [14]. Therefore, these recently characterized processes, involving either genetic compensation responses triggered by indel-derived nonsense mutations or indels as such, have the potential of hindering the creation of robust gene knockout phenotypes and predictable gene editing outcomes. For a more thorough and predictable removal of pre-existing genetic information, so-called multiplexing gene editing approaches can be deployed instead. In this case, two different programmable nucleases work in concert to generate a pair of intrachromosomal DSBs that lead to the excision of the intervening DNA sequence after end-to-end NHEJ ligation of the chromosomal termini [9,15,16,17]. Alternatively, two programmable nucleases designed for generating a pair of inter-chromosomal DSBs can direct the assembly of specific translocations to, for instance, confirm or study the involvement of these translocations in cellular transformation events and, ultimately, cancer emergence [9,18].

Normally, knocking-in gene editing strategies encompass the delivery of programmable nucleases together with exogenous donor DNA that is inserted at the site-specific DSB via either homology-independent pathways (e.g., NHEJ) [19] or homology-directed DNA repair (HDR) [9,10,11]. Generally, HDR-mediated knock-ins are more precise than those resulting from homology-independent processes in that they lack extraneous footprints at the border between endogenous and exogenous DNA. Indeed, instead of direct exogenous-to-endogenous DNA ligations via NHEJ or MMEJ, whose junction products often contain differently sized indels or specific footprints, DSB repair through HDR is a higher fidelity process [1,20]. This process involves genetic exchange between donor and target sequences and includes extensive exonucleolytic processing of chromosomal breaks, single-strand invasions, and DNA synthesis over DSB-repairing donor templates [20]. Ultimately, these molecular interactions result in accurate “copy-pasting” of the foreign genetic information into a specific locus [9,10,11]. Yet, HDR-mediated gene editing is normally less frequent than gene editing based on DNA repair mechanisms that are independent of large tracts of homology between target and donor DNA templates (e.g., cNHEJ and MMEJ). In fact, as aforementioned, cNHEJ is the main DSB repair mechanism in mammalian cells [1,20]. Further contributing to the differences in knocking-in frequencies obtained through gene editing involving cNHEJ versus HDR is the fact that the former pathway is active throughout the cell cycle; whereas the latter is only operative during the S and late G2 phases, when normally sister chromatids are available as sources of endogenous DNA-repairing templates [1,20]. For this reason, gene editing involving the recruitment of the HDR pathway is unsuitable in non-cycling cells, such as, quiescent human hematopoietic stem cells (hHSCs) and terminally differentiated cells. Another consideration concerns the steep decline in HDR-mediated gene editing frequencies as the length of the exogenous DNA increases and the extent of continuous homology between target and donor DNA decreases [21]. Therefore, the choice of the DSB repair pathway to exploit, and hence the designing of the DSB-repairing substrates to use, is contingent upon the specific application(s). For instance, knocking-in large genetic payloads into introns of safe harbor loci (e.g., *AAVS1* and *CCR5*) for achieving stable and homogeneous transgene expression in cell populations may be best pursued via selecting HDR-independent gene editing strategies; whereas knocking-in donor DNA into coding sequences for modeling or repairing genetic defects in stem or progenitor cells is best accomplished through precise HDR-dependent gene editing.

### 1.2. The Main Programmable Nuclease Platforms and Their Key Pros and Cons

Under regular conditions, HDR-mediated gene knock-ins are very rare events in human cells, with typical frequencies varying between 10^−6^ and 10^−7^ [22,23,24]. The finding that site-specific DSBs made by homing endonucleases at chromosomally embedded recombinant sequences could stimulate HDR by several orders of magnitude, was a powerful stimulus for the development of programmable nucleases [25,26,27].

The crucial feature of programmable nucleases is their capability of binding to and cleaving at predefined DNA sequences, including those located within large genomes [9,10,11,28]. Nowadays the main classes of programmable nucleases are, in chronological order of appearance, zinc-finger nucleases (ZFNs) [29], transcription activator-like effector (TALE) nucleases (TALENs) [30,31,32,33,34], and RNA-guided nucleases (RGNs) [35,36,37,38]. Naturally, the development of programmable nuclease technologies was invariably grounded on fundamental insights obtained from a broad range of biological systems, spanning from vertebrate cells and phytopathogenic bacteria, in the case of ZFNs [39] and TALENs [40,41], respectively, to bacteria and archaea, in the case of RGNs [42,43].

**Figure 1:**
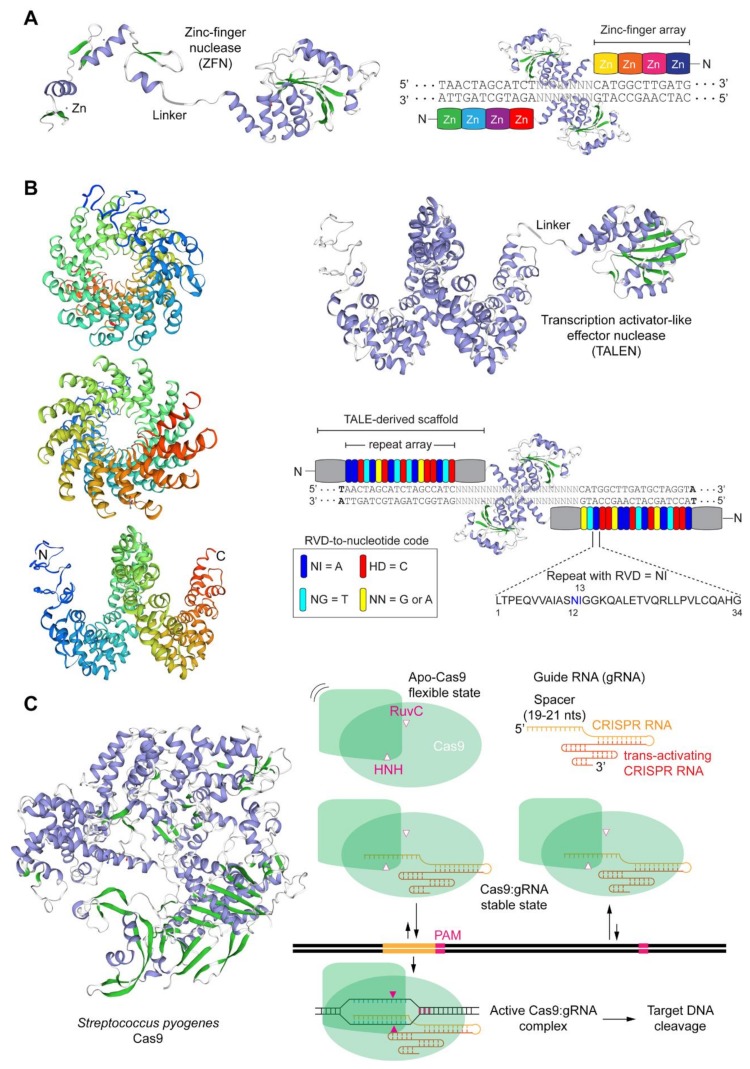
Schematics of the main programmable nuclease platforms. (**A**) Zinc-finger nucleases (ZFNs). ZFNs are chimeric modular DNA-binding proteins consisting of the FokI nuclease domain fused through a flexible linker to an array of 3–6 artificial Cys_2_-His_2_ zinc-finger motifs. Each zinc-finger motif acquires its structure through tetrahedral coordination of 2 cysteines in β-sheets and 2 histidines in α-helixes by zinc ions. ZFN monomers of a working ZFN pair bind on opposite DNA strands in a tail-to-tail configuration leading to local FokI nuclease domain dimerization and ensuing site-specific double-stranded DNA breaks (DSB) formation within the spacer sequence. (**B**) Transcription activator-like effector (TALE) nucleases (TALENs). TALENs are chimeric modular DNA-binding proteins comprising the FokI nuclease domain fused through a flexible linker to a series of typically 17.5 repeats derived from TALE proteins. TALE proteins contain a translocation and transcriptional activation domain separated by a central array of typically 33-35 isomorphic repeats. The repeats harbor at amino acid positions 12 and 13 highly polymorphic residues named repeat variable di-residues (RVDs) that bind to specific nucleotides. The structure of 17.5 TALE repeats from an engineered TALEN monomer are depicted in frontal and lateral views. TALEN monomers of a working TALEN pair bind on opposite DNA strands in a tail-to-tail configuration resulting in local FokI nuclease domain dimerization and ensuing site-specific DSB formation within the spacer sequence. (**C**) RNA-guided CRISPR-Cas9 nucleases. Engineered CRISPR-Cas9 nucleases are sequence-specific ribonucleoprotein complexes consisting of a Cas9 protein with two nucleases domains (i.e., HNH and RuvC-like) bound to a single guide RNA (gRNA) formed by a sequence customizable CRISPR RNA (crRNA) fused to a constant trans-activating CRISPR RNA (tracrRNA) scaffold moiety to which the *S. pyogenes* Cas9 enzyme binds to. Target sequences of Cas9:gRNA complexes consist of the protospacer-adjacent motif (PAM) NGG placed next to an usually 20 nucleotide-long sequence complementary to the 5′-terminal end of the crRNA (spacer). The tertiary protein structures shown, each of which derived from the primary amino acid sequences of specific ZFN, TALE and Cas9 reagents, were homology-modeled through the SWISS-MODEL server. β-sheets and α-helixes are colored in green and violet, respectively.

ZFNs and TALENs are modular proteins that present an overall similar architecture (Figure 1A,B). In particular, they consist of a customizable DNA-binding domain fused through a flexible linker to a non-specific nuclease domain, typically that of the type IIS FokI restriction enzyme whose catalytic activity is dependent on dimerization [44]. Resulting from their comparable generic architectures, ZFNs and TALENs act in a similar fashion in that members of ZFN and TALEN pairs bind in close proximity to each other on opposite DNA strands of a bipartite target sequence leading to site-specific DSBs at the spacer region after local dimerization of the FokI nuclease domains (Figure 1A,B). The DNA-binding domains of ZFNs and TALENs consist of arrays of engineered zinc-finger motifs and TALE repeats, respectively, with each zinc-finger motif usually binding to nucleotide triplets and each TALE repeat binding to single nucleotides within their respective double-stranded target sites (Figure 1A,B). Cys_2_-His_2_ zinc-fingers are found in metazoans where they serve as motifs in RNA and DNA binding proteins whose wide roles include transcriptional and epigenetic regulation of target genes [45,46]. Native TALE proteins are found in certain phytopathogenic bacteria (e.g., *Xanthomonas* sp.) where they serve as virulence factors once injected into host plant cells via type III secretory apparatuses [47]. The binding of zinc-finger motifs to specific triplets can be substantially affected by flanking nucleotides [48]. This sequence context dependency contributes to making highly specific ZFNs a laborious task requiring complex protein engineering methodologies that may include several rounds of optimization and/or screening and selection of ZFN candidates from large zinc-finger libraries [48]. In contrast, the binding of TALE repeats to their cognate nucleotides does not seem to be substantially influenced by neighboring sequences [49]. This limited sequence context dependency aids the assembly of functional and highly specific TALENs whose designing flexibility and genomic space coverage is superior to that of ZFNs [49]. DNA binding of TALEs are, however, significantly hindered by cytosine methylation [50,51] and Krüppel-associated box-induced heterochromatin [52]. Importantly, the former epigenetic modification can be elegantly surpassed by incorporating non-canonical TALE repeats within TALE arrays [51].

Native RGNs are found in many bacteria and archaea where they form adaptive immune systems against invading agents, e.g., bacteriophages and foreign plasmids [53]. Engineered RGNs, such as those based on the prototypic clustered regularly interspaced short palindromic repeat (CRISPR) and CRISPR-associated 9 (Cas9) system, from *Streptococcus pyogenes* [35,36,37,38], operate differently from ZFNs and TALENs in that target DNA cleavage does not depend exclusively on protein-DNA binding but also on RNA-DNA hybridization. In particular, RGNs, consisting of a sequence-specific single guide RNA (gRNA) coupled to an invariant nuclease, first recognize so-called protospacer adjacent motifs (PAMs) on the DNA via PAM-interacting domains in the nuclease component [10,54]. In the case of the *S. pyogenes* Cas9 the PAM reads NGG. Typically, in instances in which the 19-21 deoxyribonucleotides “upstream” from the PAM are complementary to the 5′ end the gRNA, DSB formation ensues through the concerted action of the HNH and RuvC-like nuclease domains of Cas9 (Figure 1C). The events leading to DSB formation upon initial Cas9-PAM interrogation include, PAM-proximal DNA unwinding, R-loop formation and expansion via increasing gRNA:DNA annealing which subsequently triggers HNH translocation and pairing with the RuvC-like domain. Ultimately, HNH-RuvC pairing catalyzes phosphodiester bond cleavage of both DNA chains, predominantly three base-pairs upstream from the PAM (Figure 1C) [10,43,55].

Crucially, RGNs can cut DNA at unintended genomic sequences (off-target sites) especially if mismatches between gRNA and DNA sequences locate at PAM-distal positions [56,57,58,59,60]. Furthermore, albeit to a lesser extent than NGG, *S. pyogenes* Cas9 can also effectively engage non-canonical PAMs (e.g., NAG), which further contributes to off-target activities [57,60,61]. Therefore, similarly to their programmable nuclease predecessors, the application of RGNs warrants careful assessment of potential off-target sites, especially if directed toward clinical testing. Indeed, judiciously chosen gRNAs can, per se, greatly reduce off-target activities in vitro and in vivo [62,63]. As TALENs, targeted DNA cleavage by RGNs is also hindered to some extent by epigenetic mechanisms underpinning specific heterochromatic states [52,64,65,66]. However, in contrast to TALENs, RGNs do not seem affected by DNA methylation [57].

The fact that readdressing RGNs to new target sites simply comprises modifying the 5′ end of the gRNA component, and hence does not require de novo protein engineering as ZFNs and TALENs do, confers these CRISPR-based nucleases with unsurpassed versatility and ease-of-use. Such features have fueled the primacy of RGNs amongst current programmable nuclease platforms. In fact, since the initial adaptation of natural CRISPR-Cas9 systems into genome engineering tools [35,36,37,38], RGN technologies are diversifying, being combined and adapted, at increasing rates [67]. For instance, structure-guided rational design and directed evolution approaches are producing new Cas9 variants whose features include; recognition of alternative PAMs that broaden the range of targetable genomic sites and improved target site specificities [67]. In parallel with these developments, phylogenetic analyses and mining of metagenomic datasets are unearthing components that make-up the highly diverse universe of CRISPR systems which, in addition to DNA, also target and degrade invading RNA [53]. Many of these components end up being successfully converted into reagents for (epi)genome and transcriptome modification or modulation in mammalian cells [67,68,69].

### 1.3. A Brief Overview on the Biology of Adenoviruses and Their Recombinant Types

**Figure 2:**
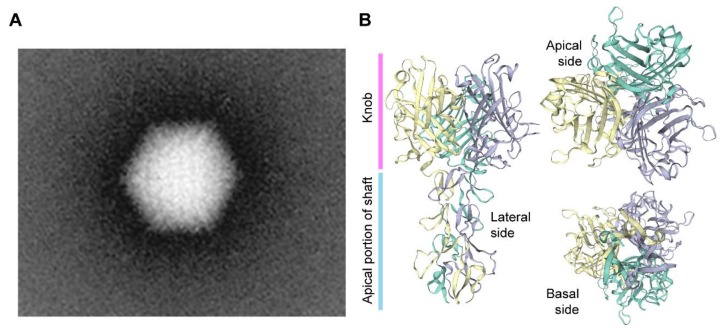
Adenovirus particle and the structure of its cell receptor-interacting fibers. (**A**) Transmission electron microscopy image of an adenovirus particle (virion). The icosahedral shape of the non-enveloped virion capsid can be discerned (~90 nm). A few of the twelve slender protruding fibers with their apical globular knob domains responsible for the initial interaction with the host-cell coxsackie and adenovirus receptor (CAR), can equally be discerned. (**B**) Three-dimensional model of the apical regions of the adenovirus serotype 5 fiber. The fiber is a homotrimer of the polypeptide encoded by the *L5* open reading frame and consists of the tail (not shown), the rod-like shaft and the globular knob domains. The tail anchors the fiber to the adenovirus capsid via non-covalent binding to the penton base proteins; the shaft projects the knob away from the capsid facilitating its interaction with CAR on the surface of host cells. The quaternary protein structure was homology-modeled using the SWISS-MODEL server and is depicted in different angles.

Adenoviruses are a diverse group of viruses from the *Adenoviridae* family that have been evolving in a wide range of vertebrates, including humans, where they cause mild ailments, e.g., in the respiratory and gastrointestinal tracts [70,71,72]. Human adenoviruses belong to the *Mastadenovirus* genus with over 55 different serotypes identified so far. The various serotypes are grouped in species A through G based on phylogenetic, genome structure and hemagglutination criteria. Structurally, adenoviral particles (virions) consist of a non-enveloped icosahedral protein capsid displaying protruding fibers [70,71,72] (Figure 2). A linear double-stranded DNA genome with terminal proteins bound to their 5′ ends is packaged inside each virion capsid consisting of 240 trimers of the hexon protein, 12 pentamers of the penton base polypeptide and 12 trimeric fiber proteins that protrude from each of the 12 capsid vertices (Figure 2). Each homo-trimeric fiber consists of a basal tail domain that docks within the penton base axis, a slender shaft region and an apical globular knob domain responsible for the initial attachment of the virion to host cell receptors (Figure 2). In addition to the major capsomers hexon, penton base, and fiber, the adenoviral capsid also contains other so-called minor proteins some of which are thought to be important for cementing the virion structure [72,73]. Adenovirus serotypes present broad cellular tropisms owing to their usage of a wide range of cell surface receptors. Identified primary attachment receptors include, the coxsackie and adenovirus receptor (CAR) used by the prototypic serotypes 2 and 5 from species C [74,75] and CD46 and desmoglein-2 engaged by species B serotypes [76,77]. Certain serotypes engage instead glycans and polysialic acids as primary attachment moieties [78,79]. The natural diversity of adenoviruses and their corresponding wide range of host-cell receptors is permitting; (i) constructing new vectors based on rare serotypes that can escape pre-exiting immunity to adenoviruses prevalent in the human population, for anti-cancer and vaccination purposes [80]; and (ii) changing the tropism of established vectors based on species C adenovirus serotype 5 into those of other serotypes so that cells with therapeutic relevance lacking CAR can be efficiently transduced [81]. For instance, genetic retargeting of vector particles through the exchange of the apical regions of the adenovirus serotype 5 fiber (Figure 2) for those of species B adenovirus serotype 35 or 50 permits efficient transduction of CAR^low^/CD46^high^ hHSCs [82,83], human mesenchymal stromal cells (hMSCs) [84,85] and human muscle progenitor cells [86].

The processes through which adenoviruses introduce their genomes into host-cell nuclei have been most extensively studied in the case of serotype 5 [87]. Briefly, after the initial attachment to the host cell, endocytosis via clathrin-coated vesicles is triggered by interactions between RGD motifs in penton bases and cellular integrins (e.g., α_v_β_5_). Subsequently, incoming fiberless virions escape lysosomal degradation via the lowering of the pH in endosomes that permits remodeled capsid components to lyse the vesicle membranes. Once in the cytosol, the remodeled nucleocapsids bind to motor proteins dynein/dynactin that transports them along the microtubule network until they dock at the nuclear pore complex and release the packaged DNA into the nucleoplasm [87].

**Figure 3:**
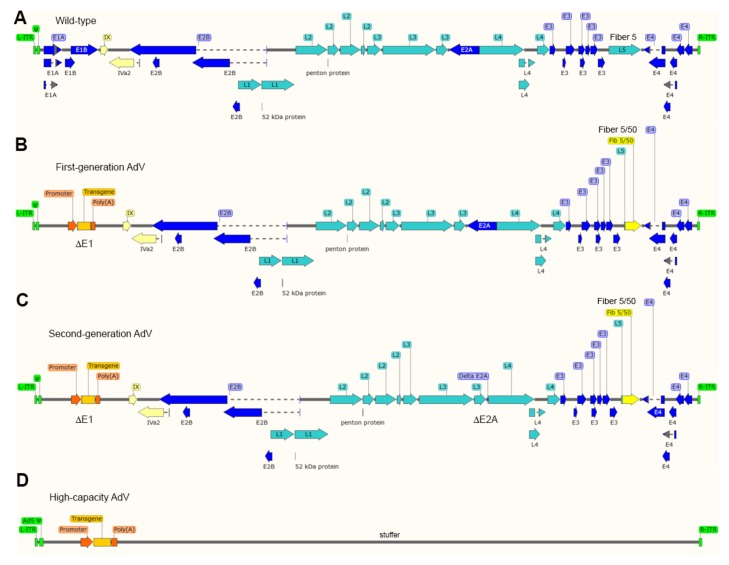
Schematics of wild-type and recombinant adenoviruses. (**A**) Genome structure of the prototypic human adenovirus serotype 5 drawn in relation to the genome structures of (**B**) first-generation (*E1*-deleted), (**C**) second-generation (*E1*- and *E2A*-deleted), and (**D**) third-generation or high-capacity (fully viral gene deleted) adenoviral vectors. The vectors contain a typical expression unit (transgene) consisting of a coding sequence of interest under the transcriptional control of a heterologous promoter and a polyadenylation signal. The first- and second-generation vector genomes encode chimeric fibers consisting of the basal shaft sequence of the human adenovirus serotype 5 linked to the apical shaft and knob domains from the CD46-interacting human adenovirus serotype 50 (yellow arrows). The non-coding *cis*-acting elements involved in vector genome replication and encapsidation are the inverted terminal repeats (ITRs) and packaging signal (Ψ), respectively. The latter signal and the “left” and “right” ITRs (L-ITR and R-ITR, respectively) are depicted in green. Regulatory functions necessary for activating the viral gene expression program are encoded by the early (E) regions *E1A*, *E1B*, *E2A*, *E3* and *E4* (dark blue arrows). The structural proteins required for assembling mature virions are encoded by the late (L) regions *L1* through *L5* (light blue arrows). The *L5* open reading frame (ORF) yields the cell surface receptor-interacting fibers. The full activation of the late viral gene expression program takes place after the onset of viral DNA replication. The ORFs coding for the intermediate proteins IX and IVa2 are also shown (light yellow arrows). Other adenoviral ORFs, e.g., small non-coding RNAs VAI and VAII are not depicted. The SnapGene software (version 5.0.7) was used for generating the different diagrams on the basis of the human adenovirus serotype 5 source sequence retrieved from GenBank accession number: AY601635.1.

The most thoroughly used adenoviral vectors (AdVs) are deleted in the transcriptional units *E1A* and *E1B* that make-up the early region 1 (*E1*) (Figure 3). The production of these first-generation, *E1*-deleted, AdVs takes place in packaging cell lines (e.g., HEK293 and PER.C6) that express, and hence complement, *in trans* the *E1* gene products [88,89]. The deletion of *E1*, firstly, blunts the activation of the regular adenoviral gene expression program preventing the replication of vector particles in transduced cells and, secondly, creates room for the packaging of approximately 5.0 kb of exogenous DNA in adenoviral capsids. Since the *E3* region is dispensable for replication in cell culture systems, some vector designs combine deletions in *E1* with deletions in *E3* that permit the packaging of up to 8 kb of exogenous DNA [71]. As it came to be known, the *E1* deletion does not fully prevent residual expression from some of the transcriptional units that remain in vector genomes [71]. The resulting leaky synthesis of viral gene products leads to vector dose-dependent cytotoxicity in vitro and short-lived transgene expression in vivo (2-3 weeks) due to the clearance of transduced cells by the immune system [90]. For this reason, *E1*-deleted AdVs, in particular those based on serotypes with low seroprevalence in the human population, are being applied in clinical trials not for gene therapies requiring prolonged transgene expression but as vaccination agents instead, e.g., against hemorrhagic fever and AIDS caused by Ebola and HIV-1 infections, respectively [91,92].

Second-generation AdVs combine deletions in *E1* or *E1* and *E3* with deletions in other early regions, i.e., *E4* or *E2* (Figure 3). Therefore, these vectors are generated in specialized packaging cell lines that complement *in trans* the respective missing gene products [71]. Although second-generation AdVs are more crippled than first-generation AdVs, at high vector doses, leaky synthesis of viral gene products can still be detected which also correlates with short-term transgene expression in vivo [71,93].

To abrogate altogether leaky viral gene expression in transduced cells and, at the same time, maximize the size of foreign DNA that can be incorporated in adenoviral capsids, high-capacity adenoviral vectors (HC-AdVs) were developed [71] (Figure 3). These third-generation AdVs (a.k.a. “gutless” or helper-dependent AdVs) lack all viral coding sequences retaining from the parental virus genome exclusively the short *cis*-acting inverted terminal repeats (ITRs) (103-bp each) and packaging elements needed for, respectively, vector DNA replication and encapsidation in producer cells (Figure 3). The need for complementing *in trans* the full set of adenoviral gene products, makes the production of HC-AdVs more complex than that of their earlier generation counterparts. In particular, HC-AdV particles are assembled in *E1*-complementing cell lines that express a site-specific recombinase (e.g., Cre or FLP) [71,94,95]. These producer cell lines are transduced with an *E1*-deleted helper AdV that expresses *in trans* the viral gene products necessary for the replication and packaging of HC-AdV genomes into adenoviral capsids. Crucially, the packaging signals of the helper genomes are flanked by recognition sequences for the site-specific recombinase so that the vast majority of assembled AdV capsids contain HC-AdV DNA in detriment of helper DNA owing to the selective recombinase-mediated removal of the packaging elements from the latter templates. Normally, besides the adenoviral *cis*-acting elements and the foreign DNA of interest, HC-AdV genomes also contain a so-called “stuffer” DNA segment to increase the HC-AdV DNA length to at least ~28 kb and, in doing so, guarantee vector genome stability during replication in producer cells [94]. 

## 2. Adenoviral Vector-Based Gene Editing in Human Adult Stem Cells and Their Progeny

### 2.1. Targeted Gene Disruption

Various viral vector systems initially developed for transgene expression and gene therapy undertakings, have also started to be investigated and coopted as gene editing agents (for a review on their features and main pros and cons, see, ref. 9). In fact, all three classes of replication-defective AdV systems (Figure 3) are included in these gene-editing research efforts, that are covered next.

*E1*-deleted AdVs based on serotype 5 displaying apical fiber motifs from CD46-interacting serotype 35 (AdV5/35) have been tested for conferring resistance to HIV-1 infection. In particular, AdV5/35 vectors encoding *CCR5*-specific ZFNs were applied for NHEJ-mediated generation of human CD4^+^ T cells with reduced amounts of the transmembrane HIV-1 co-receptor protein CCR5 [96]. The ex vivo cell transduction protocol resulted in 40–60% disruption of *CCR5* alleles in these cells. Importantly, transplantation experiments in immunodeficient NOD/Shi-scid/γc^−/−^ (NOG) mice led to a 3-fold enrichment of CD4^+^ T cells with *CCR5* knockout alleles in animals infected with HIV-1, suggesting selection for gene-modified cells. Notably, next generation sequencing (NGS) analysis of transduced CD4^+^ T cells revealed a substantial ZFN-induced off-target activity (i.e., 5.39% indels) at the neighboring and highly sequence identical *CCR2* locus [96]. Building on this principle but aiming at a longer protective effect against HIV-1 infection, another study focused on targeting adult hematopoietic stem/progenitor cells (HSPCs). In this work, AdV5/35-mediated delivery of *CCR5*-specific ZFNs into HSPC-enriched CD34^+^ cells led to target allele knockout frequencies above 25%. However, these knockout levels were only obtained in the presence of protein kinase C (PKC) activators, an expedient used to presumably improve vector transduction and/or ZFN expression [97]. Moreover, low, yet detectable, off-target activity at *CCR2* and at three other non-coding sequences located elsewhere in the genome were observed via NGS analysis. Subsequent cell transplantation assays in immunodeficient NOD/SCID/γc^−/−^ (NSG) mice showed a vector dose-dependent reduction in the levels of human cell engraftment as measured by CD45^+^ cell counts in animals infused with HSPCs treated with PKC activators and *CCR5*-targeting ZFNs [97]. To avoid the toxicity caused by PKC activators, Maier and co-workers tested instead anti-CD3/CD28 stimulation as an adjuvant for improving transduction of T lymphocytes by an AdV5/35 vector encoding *CCR5*-specific ZFNs [98]. When compared to the experimental group exposed to PKC activation, this method enhanced the frequencies of target gene knockout by almost 3-fold (up to 32%). Importantly, ZFN-associated toxicity was not detectable with levels of off-target *CCR2* disruption in transduced T lymphocytes remaining below 4%, as estimated through genotyping assays based on mismatch-sensing nucleases and DNA fluorescence densitometry [98].

The generation of AdVs encoding ZFNs is challenging due to cytotoxicity caused by transgene overexpression in producer cells. To overcome this limitation, Saydaminova and colleagues exploited miRNA-dependent downregulation of transgene expression in 293-Cre packaging cells. This strategy permitted generating tropism-modified HC-AdVs encoding *CCR5*-specific ZFNs at high yields and without vector genome rearrangements. Importantly, miRNA profiling guaranteed that the endogenous miRNA suppressing ZFN synthesis in producer cells was not expressed in hHSC-enriched CD34^+^ target cells [99]. Transduction of the erythroleukemia cell line MO7e and primary CD34^+^ cells with the resulting HC-AdV coding for the *CCR5*-specific ZFNs led to 43.6% and 13% indel formation, respectively, at *CCR5* as determined by mismatch-sensing nuclease assays. Cell transplantation experiments in immunodeficient NOG mice revealed, however, that human CD34^+^ cells transduced with the ZFN-encoding HC-AdV engrafted in the bone marrow at 3-fold lower levels than their non-transduced counterparts (i.e., 2.12% versus ~6%, respectively) [99].

A *CCR5*-specific ZFN pair delivered ex vivo into autologous CD4^+^ T cells of AIDS patients by an *E1*-deleted AdV5/35 vector formed the basis for the first clinical testing of a programmable nuclease [100]. The infusion of 10 billion cells, of which 11–28% were *CCR5*-disrupted, was shown to be safe. Moreover, edited cells persisted after transplantation with a mean half-life of 48 weeks and, tantalizingly, upon an interruption of anti-retroviral therapy, the rates with which *CCR5*-disrupted cells declined were significantly slower than those of unmodified cells [100]. Outstanding questions following from this landmark study are the feasibility in achieving sufficient numbers of cells with bi-allelic *CCR5* knockout without inducing cytotoxicity and with minimal ZFN-induced off-target effects. Finally, the combination of genetically retargeted AdV5/35 vectors and ZFN technologies has also been used for knocking out endogenous T-cell receptor genes and the primary HIV-1 receptor gene *CXCR4* in T cells [101,102]. 

In addition to ZFNs, the AdV platform is equally suitable for the delivery of TALENs into human somatic cells, e.g., muscle progenitor cells and hMSCs. In fact, Holkers and co-workers demonstrated that, in contrast to HIV-1-based lentiviral vectors, transgenes encoding TALENs can be transferred intact into human cells by AdVs [103]. Indeed, lentiviral vectors encoding TALENs suffer substantial genetic rearrangements in the form of deletions of various sizes that occur within the direct repeats corresponding to the TALE DNA-binding domains (Figure 1B). These deletions are likely caused by frequent reverse transcriptase template switching events taking place within the TALE repetitive tracts. Thus, the transfer of transgenes coding for TALE-based proteins through standard and integration-defective lentiviral vectors (IDLVs) requires substantial coding sequence optimization for minimizing sequence identity among repeats [104,105]. 

It is also noteworthy to mention that, although IDLVs permit transient expression of ZFNs and sequence optimized TALENs in human cells, the yields necessary for robust targeted DSB formation might not be reached due to epigenetic silencing mechanisms directed at IDLV genomes involving histone deacetylases [106,107]. In contrast, functional assays revealed that AdVs expressing TALENs allow for robust targeted DSB formation in several human cell types, e.g., muscle progenitor cells and hMSCs [103]. Second-generation AdVs deleted in *E1* and *E2A* and displaying apical motifs from CD46-interacting serotype 50 (AdV5/50) were used in these proof-of-concept experiments validating the AdV platform for the delivery of functional TALENs into human cells [103]. Follow-up experiments using first-generation and second-generation fiber-modified AdVs encoding TALENs and *S. pyogenes* Cas9 addressed to sequences flanking the major *DMD* mutational hotspot triggered large deletions comprising multiple exons (>500 kb) in patient-derived muscle progenitor cells [17]. These maneuvers designed for repairing *DMD* alleles causing Duchenne muscular dystrophy (DMD), led to the synthesis of in-frame mRNA transcripts encoding a truncated yet potentially functional Becker-like dystrophin protein [17].

Currently, the integration of AdV and programmable nuclease technologies for gene editing in somatic cells is dominated by the delivery and testing of RGNs. The first viral vector-mediated delivery of RGN components into mammalian cells consisted of using fiber-modified *E1*- and *E2A*- deleted AdVs expressing Cas9 or gRNAs directed to either a chromosomally integrated *EGFP* reporter or to the *AAVS1* safe harbor locus located in the human chromosome 19 at position 19q13.3-qter. In co-transduction experiments, robust targeted DSB formation was achieved at *AAVS1* in several cell types including human muscle progenitor cells and hMSCs [108]. In another study, co-transduction of human lung microvascular endothelial cells with an *E1*-deleted AdV and a lentiviral vector encoding Cas9 and a *TIE2*-specific gRNA, respectively, induced up to 90% of target gene disruption. Direct phenotypic analysis of *TIE2*-edited cell populations showed a persistent increase in endothelial cell permeability when compared to control cells [109]. 

In addition to NHEJ-mediated target gene disruption for basic biology studies, AdV-mediated RGN delivery is also being explored for modifying genes underlying human disorders. In this regard, to facilitate the delivery of RGN components, Maggio and colleagues co-packaged Cas9 and gRNA expression units within single particles of fiber-modified *E1*- and *E2A*-deleted AdVs [17]. In these experiments, testing “all-in-one” AdV-mediated transfer of RGN components, *DMD* exons 51 and 53 were separately targeted for resetting the *DMD* reading frame in muscle progenitor cells derived from DMD patients [17]. In a follow-up study, fiber-modified *E1*- and *E2A*-deleted AdVs encoding Cas9 and gRNA pairs targeting *DMD* introns 52 and 53 or introns 43 and 54 were assembled for triggering single or multiple exon deletions, respectively [110]. The latter dual RGN-encoding vector permitted removal of the aforementioned major *DMD* mutational hotspot in up to 18% of target alleles in patient-derived muscle progenitor cells [110]. More recently, fiber-modified HC-AdVs were applied for the delivery of optimized high-specificity dual RGNs equally targeting *DMD* introns 43 and 54. The transduction of muscle progenitor cells isolated from DMD patients with these CD46-targeting HC-AdV particles resulted in the removal of the major *DMD* mutational hotspot in up to 42% of target alleles resulting in the direct detection of Becker-like dystrophin synthesis in differentiated muscle cell populations [111]. 

A study by Li and coworkers documented over 30% indel formation at *CCR5* in CD4^+^ T cells that had been pretreated with a PKC activator and subsequently selected for RGN expression after exposure to *E1*-deleted AdV5/35 particles encoding EGFP-tagged RGNs. Significantly, the authors obtained evidence for the acquisition of resistance of *CCR5*-edited CD4^+^ T cells to two different HIV-1 strains in vitro [112].

Disruption of binding motifs for the *HBG* repressor protein BCL11A is a promising strategy to reactivate *HBG* expression and fetal γ-globin synthesis to complement the absence of functional adult β-globin in β-thalassemic and sickle cell disease (SCD) patients. In this regard, transduction of mobilized peripheral blood CD34^+^ cells from healthy donors with fiber-modified HC-AdVs encoding *HBG*-specific RGNs led to around 20% of target motif disruption in these cells [113]. Moreover, no indels were observed in the top 10 candidate off-target sites, as assessed by mismatch-sensing nuclease assays and, importantly, the erythroid differentiation capability of the gene-edited hematopoietic progenitors was maintained [113]. Cell transplantation assays in lethally irradiated immunodeficient mice revealed indel frequencies ranging from 19% to 25% at *HBG* alleles in human CD45^+^ cells isolated from bone marrow at 10 weeks post-transplantation. Upon in vitro differentiation of these bone marrow-derived CD45^+^ cells, the frequencies of γ-globin^+^ cells were ~50% and ~27% in the transduced and non-transduced groups, respectively, as determined by flow cytometry [113]. In addition, β-YAC/CD46 mice were also used in this study to overcome the known block on human erythrocytic lineage differentiation in NSG mice. β-YAC/CD46 mice contain a human DNA fragment encompassing the entire 82-kb human β-globin locus and express the human CD46 receptor which permits transducing mouse cells with HC-AdV particles displaying adenovirus serotype 35 fibers. Hence, this mouse model allows in vivo evaluation of *HBG* reactivation in mature circulating erythrocytes. Bone-marrow Lin^−^ cells isolated from β-YAC/CD46 mice were transduced with the fiber-modified HC-AdVs encoding *HBG*-specific RGNs and were subsequently transplanted into lethally irradiated C57BL/6 recipient mice. At 10 weeks post-transplantation, there was a ~5-fold reduction of *HBB* mRNA and a ~30-fold increase in *HBG* mRNA levels in red blood cells when compared to controls. These results indicate that a switch in the balance of adult to fetal globin expression was achieved [113]. In another study, Li and co-workers using fiber-modified HC-AdVs encoding RGNs targeting *BCL11A* gene enhancer or BCL11A protein binding sequences obtained over 20% indel formation at these motifs in CD34^+^ cells [114]. Interestingly, however, in vitro colony-forming unit (CFU) assays based on semi-solid methyl-cellulose medium showed a reduction in the number of multi-lineage progenitors derived from vector-transduced cells [114]. In addition, cell transplantation assays in irradiation-conditioned NSG mice demonstrated that engraftment rates of CD45^+^ cells in mice receiving grafts transduced with RGN-encoding HC-AdVs were 5- to 10-fold lower than those transplanted with non-transduced cells or cells transduced with a control vector encoding exclusively Cas9 [114]. The low numbers of CFUs in vitro and engraftment rates in vivo indicated RGN-induced cytotoxic effects. In line with this data, Schiroli and colleagues found through single-cell transcriptomics analysis that DSBs induced by ZFNs and RGNs can activate a P53-dependent DNA damage response in HSPCs [115]. To shorten the duration of RGN activity, bacteriophage anti-CRISPR (Acr) peptides AcrIIA2 and A4, were exploited to inhibit long-term Cas9 activity [114]. Sequential transfer of *BCL11A* enhancer-specific RGNs and Acr peptides via tropism-modified HC-AdV transductions with an interval of 48 hours led to 37.9% indel formation in the human umbilical cord blood-derived erythroid progenitor cell line HUDEP-2 [114]. Flow cytometry and qRT-PCR analyses showed a switch of *HBB* to *HBG* expression in the edited HUDEP-2 populations. After applying a similar sequential HC-AdV transduction protocol to CD34^+^ cells followed by transplantation of vector-treated cells into irradiation-conditioned NSG mice, Li and coworkers observed comparable levels of CD45^+^ cell engraftment in mice receiving non-transduced and vector-transduced cells. Indel frequencies at the *BCL11A* gene enhancer and BCL11A protein binding site ranged from 8.5% to 27% and from 10.5% to 21%, respectively, in CD45^+^ cells isolated from bone marrow, as measured by mismatch-sensing nuclease assays. Finally, in vitro differentiation of isolated CD45^+^ cells into erythroid cells, revealed a ~1.4-fold increase in the percentage of γ-globin^+^ cells in the edited over the control groups [114].

### 2.2. Targeted Gene Integration 

As aforesaid, HDR leads to precise genomic DNA editing in the presence of exogenous donor templates that can be designed for gene knock-ins, gene knockouts or gene correction. Therefore, AdVs are also being utilized for transferring programmable nucleases together with donor templates into human cells. In this context, Coluccio and colleagues combined AdV-mediated ZFN delivery with the transfer of donor HDR substrates in AdVs or IDLVs for testing homology-directed gene insertion in human keratinocytes [116]. In this study, *AAVS1*-specific ZFNs were delivered by an *E1*-deleted AdV5/35 vector, whereas the donor, containing a reporter gene flanked by *AAVS1*-targeting homologous sequences, was transferred via either vesicular stomatitis virus glycoprotein G-pseudotyped IDLV or *E1*-deleted AdV5/50 particles. Transduction of HaCaT cells, a human keratinocyte cell line, with ZFN-encoding AdV particles together with IDLV or AdV donors led to chromosomal transgene integration frequencies of 20% and 1%, respectively [116]. However, combining AdV5/35 and IDLV vectors for introducing into human primary keratinocytes *AAVS*1-specific ZFNs and donor templates, respectively, resulted in substantially lower frequencies of stable transgene insertion (i.e., 0.3%), presumably in part due to the observed inefficient transduction of these target cells by IDLV particles [116]. In another study, investigating homology-directed gene targeting, Holkers and coworkers combined the transfer of HDR substrates in AdV or IDLV particles with AdV-mediated delivery of TALENs instead [117]. In particular, *AAVS1*-specific TALENs were delivered by an *E1*-deleted AdV5/50 vector, whereas the donor, containing a reporter gene flanked by *AAVS1*-targeting sequences, was transferred via either IDLV or *E1*- and *E2A*-deleted AdV5/50 particles. Transduction of human muscle progenitor cells with TALEN-encoding AdVs together with IDLV or AdV donors led to chromosomal transgene integration frequencies of 9.1% and 1.24%, respectively. These data together with that of Collucio and coworkers indicate that IDLV donors lead to higher frequencies of DSB-dependent gene knock-ins than those achieved by AdV donors. However, isolation of genetically modified muscle progenitor cells (n = 214 clones) followed by clonal analysis using junction PCR assays demonstrated that a large proportion of IDLV-modified cells contained random insertions (13.4%) or inaccurate *AAVS1* insertions (44.3%), of whom a substantial fraction corresponded to head-to-tail donor DNA concatemers (38.5%). In contrast, neither random insertions nor inaccurate *AAVS1* insertions were detected in the randomly isolated AdV-modified cells [117]. Thus, although free-ended IDLV genomes lead to higher frequencies of genetically modified cells than protein-capped AdV genomes, the latter genomes result in more specific and accurate HDR-mediated donor DNA insertion [28,117]. The relevance of the donor DNA structure to the specificity and accuracy of gene targeting was demonstrated by experiments in which the excision of HDR substrates from the context of protein-capped AdV genomes resulted in an increase in random donor DNA insertions, as determined by clonal analysis using junction PCR assays [117]. Presumably, albeit more efficacious for generating populations of genetically modified cells, linear free-ended DNA is prone to homology-independent capture at chromosomal DSBs (targeted or otherwise) through illegitimate recombination processes comprising end-to-end DNA ligations. 

Li and colleagues applied HC-AdV5/35 vectors for delivering into human CD34^+^ cells *AAVS1*-specific RGNs and donor DNA templates encoding EGFP and the positive selectable marker mgmt^P140K^ [118]. The latter gene product confers resistance to O6BG/bis-chloroethylnitrosourea (BCNU). In this study, *AAVS1* gRNA target sites flanked the donor template for enhancing the frequencies of genetically modified cells via RGN-induced donor DNA excision. Co-transduction of human CD34^+^ cells with both AdVs resulted in 0.9% of EGFP^+^ hematopoietic cell clones as determined by CFU assays. Further characterization of these colonies (n=14) showed accurate insertion of the donor DNA at the *AAVS1* locus. The delivery of *AAVS1*-specific RGNs and *AAVS1*-targeting donor templates into murine Lin^−^ cells, isolated from the bone marrow of human *AAVS1*/CD46 transgenic mice, was done through their ex vivo co-transduction with HC-AdV5/35 particles. As controls, parallel samples of Lin^−^ cells were exposed exclusively to one of the two vectors. Subsequently, vector-transduced Lin^−^ cells were transplanted into lethally irradiated C57BL/6 mice. Notably, in these experiments, no significant differences in engraftment rates were observed in mice receiving cells treated with the different HC-AdV5/35 regimens. At 4 weeks post-transplantation, an average of 1.1% and <0.2% of EGFP^+^ peripheral blood mononuclear cells (PBMCs) were measured in the experimental and control groups, respectively. After three rounds of BCNU selection an enrichment in EGFP^+^ cell marking was observed that varied from ~20 to ~100%, depending on the recipient mouse analyzed. Importantly, multilineage EGFP^+^ cell marking was stably maintained for 16 weeks in secondary recipients demonstrating genetic modification of *bona fide* murine HSCs. Building on these data and experimental settings, Li and colleagues went on to test HDR-mediated knock-in of a γ-globin-coding transgene at the human *AAVS1* locus in murine Lin^−^ cells isolated from AAVS1/CD46 transgenic mice. The transgene was placed under the regulation of a mini-β-globin locus control region for preferential expression in erythroid cells. Lin^−^ cells transduced with HC-AdV5/35 particles were transplanted into lethally irradiated C57BL/6 mice and were subsequently subjected to three rounds of BCNU selection. At 16 weeks post-transplantation, the level of γ-globin was on average 20.52% and 22.33% of that of adult mouse β-globin as measured by high-performance liquid chromatography and qRT-PCR analyses, respectively [118]. 

The cumulative data from these investigations on the use of AdV systems for gene editing of adult stem cells and their progeny bodes well for their application in basic research and biotechnologies, including for the development of genetic therapies targeting acquired and inherited disorders.

## 3. Human Embryonic Stem Cells (hESCs) and Human Induced Pluripotent Stem Cells (hiPSCs) Genome Editing

**Figure 4:**
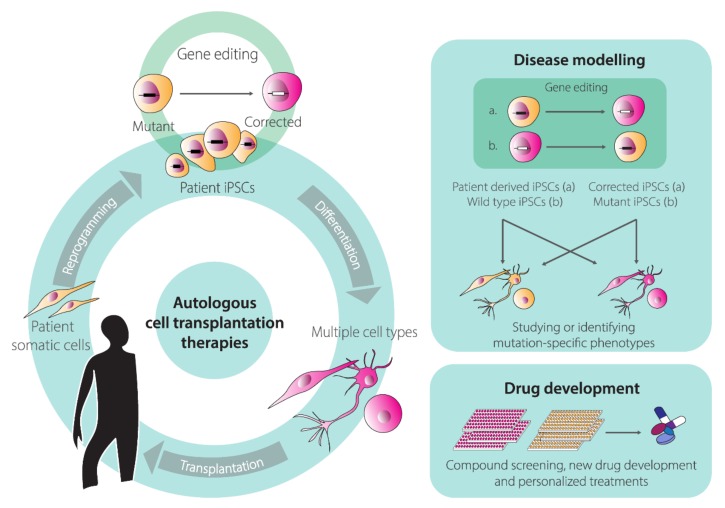
Illustration of human induced pluripotent stem cell (hiPSC)-based research and development activities enabled by genome editing technologies. Ex vivo reprogramming of patient-derived somatic cells into hiPSCs followed by their genetic correction, expansion, and directed differentiation into specialized cells types opens the perspective for the development of innovative autologous cell therapies. Generation of hiPSC lines sharing the same genetic background and differing from each other at predefined genetic loci can be accomplished via either (**a**) targeted correction of specific mutations in patient-derived hiPSCs or (**b**) targeted installation of specific mutations in wild-type, healthy donor-derived, hiPSCs. The resulting pairs of isogenic hiPSC lines form tractable experimental systems for the controlled and robust establishment of genotype-phenotype associations during disease modeling and for high-throughput screens aiming at assessing drug toxicities and/or identifying new drug candidates.

Human pluripotent stem cells (hPSCs) renown rose ever since the first isolation of human embryonic stem cells (hESCs) from pre-implantation embryos in 1998 [119]. Under well-defined culture conditions, hESCs are able to self-renew and can replicate for long periods in vitro while maintaining their full potential to differentiate into any somatic cell type derived from the three embryonic germ layers; endoderm, ectoderm, and mesoderm. These unique features of self-renewal and pluripotency facilitate studying cell differentiation processes and creating in vitro models of human disorders (“disease-in-a-dish”). In addition, hESCs hold the promise of revolutionizing regenerative medicine through the establishment of innovative stem cell therapies and represent invaluable tools for drug screening and development. Nevertheless, the therapeutic application of hESCs is limited not only by technical challenges but also ethical concerns stemming from their human-embryo origins [120]. For this reason, the generation of human induced pluripotent stem cells (hiPSCs) represented a fundamental turning point in this field of biomedical research [121]. This revolutionizing discovery took place in 2006, when Takahashi, Yamanaka and colleagues discovered that a cocktail of four transcription factors (i.e., KLF4, c-MYC, OCT4, and SOX2) was capable of reprogramming somatic, terminally differentiated cells, “back” to an hESC-like state [122,123]. Indeed, for the most part, hiPSCs maintain the characteristics of hESCs, including their defining features of self-renewal and pluripotency. Crucially, cellular reprogramming overcomes the ethical concerns associated with hESCs and offers the possibility for generating and differentiating hiPSCs from virtually any individual into tissue-specific cell types. These capabilities permit in vitro disease modeling and drug screenings [124,125]. Moreover, hiPSCs open the perspective for autologous cell transplantation therapies for repairing tissues and organs affected by injuries or, when combined with gene-editing technologies, inherited disorders [124,125] (Figure 4). Indeed, the advances made in gene editing technologies are greatly impacting hPSC-based research [126]. Firstly, gene editing of hiPSCs is an important steppingstone towards their clinical translation, in that targeted correction of patient-derived hiPSCs might pave the way for the development of personalized regenerative medicines of otherwise untreatable genetic diseases [126] (Figure 4). Secondly, gene editing contributes to the establishment of clear genotype-phenotype associations by permitting the generation of isogenic pairs of hiPSC lines that share the same genetic background and differ exclusively in specific well-defined DNA sequences. These isogenic hiPSC pairs can be obtained either via correcting a genetic defect in a patient-derived hiPSC line or introducing mutations causing a genetic defect in a wild-type hiPSC line (Figure 4). 

Several studies employing engineered ZFNs, TALENs, and RGNs, have shown the utility of these molecular tools for gene editing in hPSCs [127]. The off-target effects and unpredictable genomic changes resulting from the repair of DSBs made by programmable nucleases are, however, major concerns in the gene editing field, especially in its application to stem cells [56,57,58,59,60]. In this regard, recent developments on genome engineering strategies based on sequence- and strand-specific nucleases (nickases) as such [61,128,129,130] or on the fusion of these nickases to cytidine or adenine deaminases (i.e., base editors) [131] or reverse transcriptases (i.e., prime editors) [132] is gaining momentum. In part, this momentum derives from the fact that these tools open up the perspective for efficient, DSB-free, genetic modification of stem cells whose sensibility to DSBs is particularly acute [115,133,134]. Next to gene editing strategies based on nucleases and nickases, there are also gene editing approaches that rely on the exclusive delivery of exogenous HDR substrates into hPSCs. In this case, stringent positive and negative selection schemes are often necessary for the isolation of properly targeted cells as HDR events are very rare in the absence of DSBs at target DNA [25,26,27] or SSBs at target and donor DNA [61,128,129,130,135]. Moreover, to ameliorate the inefficiency of HDR in the absence of targeted DNA lesions, whenever possible, donor templates are endowed with long sequences homologous to target genomic regions. Indeed, extensive homologous sequences, normally spanning several thousands of bps flanking the desired exogenous DNA are exploited for obtaining site-specific gene insertion through spontaneous HDR. However, regardless of their dependency on or independency from nucleases or nickases, and derivatives thereof, a main challenge for operational gene editing in adult stem cells and hPSCs remains the need for delivering the necessary molecular tools in an efficient and, ideally, non-cytotoxic manner. To this end, various viral and non-viral delivery systems are being explored [9,136]. We will next highlight the contributions of HC-AdV technology for gene editing in hiPSCs and hESCs.

### 3.1. High-Capacity Adenoviral Vector (HC-AdV)-Based Gene Editing in hESCs and hiPSCs

**Figure 5:**
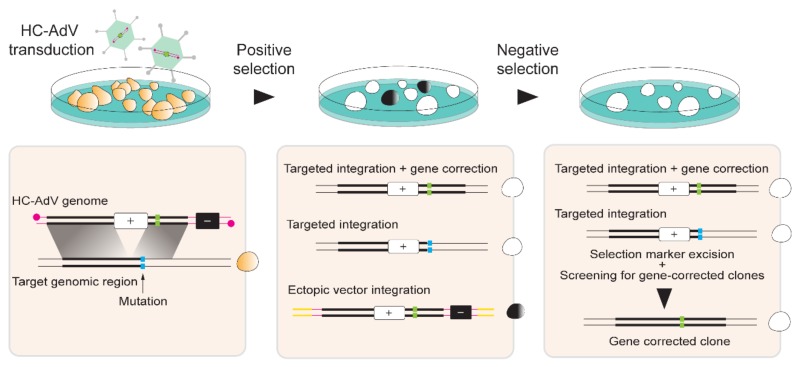
DSB-independent gene editing based on HC-AdV donor DNA transduction and positive-negative cell selection protocols. HC-AdV genomes contain a positive selection cassette, e.g., *neo*^R^ (white box) flanked by extensive human DNA sequences that are homologous to a target genomic region except for specific nucleotide(s) (left panel). In this example, donor and acceptor templates bear wild-type and mutant allelic sequences of a target gene (green and cyan boxes, respectively) so that, after recombination, involving outward homologous regions, gene correction ensues (middle panel, upper diagram). Next to these wanted outcomes there are also unwanted ones in the form of homologous and non-homologous recombination events resulting in no gene correction and random chromosomal donor DNA integration (middle panel, central, and bottom diagrams, respectively). Cells containing these different types of genetic modifications survive and multiply in the presence of a cell-killing drug that is broken-down by the positive-selection gene product. Selective elimination of cells with random HC-AdV donor DNA insertions is accomplished owing to the presence of a suicide negative selection cassette located outside the homology regions, e.g., *HSV-tk* (black box), that convers a prodrug substrate into a cell-killing product. The positive selection marker can subsequently be removed by site-specific recombinases, e.g., Cre and FLP that leave *lox*P and *FRT* site footprints, respectively, in the genome. Alternatively, transposon/transposase systems, e.g., footprint-free PiggyBac variants can be used that ultimately achieve scarless genomic modifications. Finally, genotyping screens permit identifying cells containing correctly targeted alleles (right panel).

HC-AdV-based gene editing of PSCs involving exclusively donor DNA delivery was initially applied in murine ESCs for achieving HDR-mediated correction of *Hprt* alleles [137]. Soon thereafter, Suzuki and coworkers tested HC-AdVs for gene editing in hESCs [138]. These authors started by comparing HC-AdVs displaying serotype 5 or serotype 35 fibers for transducing hESCs by measuring through flow cytometry the frequencies of cells transiently expressing the Venus fluorescent protein reporter. Both viral vectors showed a clear multiplicity of infection (MOI)-dependent increase in transduction efficiencies that reached over 90% of target cells. The highest gene transfer levels were obtained with the tropism-modified vector. Notably, at a low to moderate MOI range, i.e., 10–300 transducing units per cell (TU/cell), cytotoxic effects were not significantly different from mock-transduced cells. Subsequently, HC-AdVs displaying conventional serotype 5 fibers were employed at a MOI of 300 TU/cell to deliver an *HRPT1*-targeting construct with long regions of homology (i.e., 14.3 kb and 9.2 kb) designed to insert a *neomycin phosphotransferase* (*neo*^R^) cassette. Cells stably expressing the *neo*^R^ gene product acquire resistance to the aminoglycoside antibiotic G418 (also known as geneticin) (Figure 5). In addition to the positive selection marker gene *neo*^R^, in order to minimize the expansion of cells with ectopic vector DNA integration, the vector genome also contained a negative selection cassette external to the homology regions expressing the Herpes Simplex Virus type 1 thymidine kinase (HSV1-tk) (Figure 5). Therefore, in case of HDR-independent or random chromosomal integration of HC-AdV DNA, stable HSV1-tk synthesis converts the pro-drug ganciclovir (GCV) into a phosphorylated cytotoxic product that leads to cell death (Figure 5). Among 5.1×10^6^ transduced hESCs, 136 colonies were G418-resistant and, of these, 31 were G418/GCV double-resistant. PCR and Southern blot analyses further demonstrated that of the 31 double-resistant colonies, 14 were correctly targeted at *HPRT1* [138]. Importantly, HC-AdV transductions led to significantly higher gene transfer efficiencies than those obtained by “naked” DNA transfections based on electroporation and FuGENE HD. Moreover, when compared to the electroporation of the same *HPRT1*-targeting construct, HC-AdV donor delivery proved to be ~300 fold more efficient in terms of the frequencies of precisely edited cells obtained [138].

Building on these promising findings, a follow-up study investigated a similar HC-AdV-based gene editing approach in both hESCs and hiPSCs [139]. In this study, the authors explored different gene editing settings, i.e., (i) knock-in of a donor *neo*^R^ cassette at the housekeeping *HPRT1* locus, (ii) knock-in of a donor *neo*^R^ cassette designed for conditional knock-out of target genes located at different genomic positions, and (iii) knock-in of a donor *EGFP* cassette at a transcriptionally inactive *HB9* locus. Firstly, *HRPT1*-targeting experiments for knocking-in the donor *neo*^R^ cassette in two distinct hiPSC lines led to 20% and 7% of correctly targeted clones after positive-negative G418/GCV selection [139]. Significantly, control experiments involving the electroporation of the linearized *HPRT1*-targeting HC-AdV plasmid led to 0% of correctly targeted clones. Secondly, *neo*^R^ cassette knock-in experiments at *KU80, LIG1,* and *LIG3* led to 81%, 34%, and 42% gene targeting frequencies, respectively. Subsequently, the *lox*P-flanked *neo*^R^ cassette was excised in ~25% of the targeted cells through transient Cre delivery and target gene knockouts were confirmed through clonal analyses using Southern blotting, RT-qPCR, and western blotting. Finally, HC-AdV-mediated *EGFP* knock-in at the transcriptionally inactive *HB9* locus led to 23% and 57% of accurate gene targeting in hiPSC and hESC lines, respectively. Other studies confirmed that silent loci are accessible to HC-AdV-based gene editing. For example, to trace gene expression during cell differentiation, HC-AdVs were employed to knock-in live-cell reporter genes into *ALB* and *OC* alleles to monitor the differentiation of hESCs and/or hiPSCs along the hepatic and osteogenic lineages, respectively [140,141].

### 3.2. HC-AdV-Based Gene Editing for Targeted Gene Correction in Human Pluripotent Stem Cells (hPSCs) 

HC-AdVs are also being investigated for targeted correction of disease-causing mutations in hPSCs (Figure 5). Initial experiments targeted mutations underlying Hutchinson–Gilford progeria syndrome (HGPS) and atypical Werner syndrome (AWS) in hiPSCs [142]. HGPS and AWS are laminopathies whose mutations in the exon 11 of the *LMNA* gene include C1824T and A1733T, respectively. These mutations affect the nuclear structure resulting in premature aging. By exploiting the large cloning capacity of HC-AdV particles, HGPS and AWS can potentially be tackled by a single large *LMNA*-targeting construct covering different mutations. Similar to previous work [138], upon HC-AdV donor DNA transduction of hiPSCs and positive-negative G418/GCV selection, integration of the *neo*^R^ cassette at the *LMNA* target site between exons 10 and 11 ranged from 78% to 100%, as assessed through PCR and Southern blot analyses [142]. Correction of the 1-bp substitutions C1824T and A1733T located in exon 11 of *LMNA* in HGPS-hiPSCs and AWS-hiPSCs, respectively, was verified through DNA sequencing of targeted clones. This analysis revealed that 12 out of 25 HGPS-hiPSC clones and 35 out of 65 AWS-hiPSCs clones were accurately repaired. Subsequently, the *neo*^R^ cassette, flanked by *FRT* sites, was excised by transient expression of FLPe recombinase leading to wild-type *LMNA* expression and subsequent rescue of the HGPS phenotype, as determined by the restoration of normal nuclear architecture and cell senescence programs [142]. Next, in addition to confirming the pluripotency of gene-edited hiPSCs, the authors meticulously investigated the genetic and epigenetic integrity of the corrected cells. In particular, correctly targeted hiPSCs showed a normal karyotype, expressed pluripotency markers and exhibited demethylation of the promoter of the pluripotency gene *OCT4* [142]. Moreover, genome-wide single nucleotide polymorphism (SNP), DNA microarray, and genome-wide DNA methylation analyses indicated a generic maintenance of the genetic background, global gene expression patterns, and global epigenetic states, respectively, in gene-edited cells using parental hiPSC lines as references [142]. In another study, HC-AdV-based gene editing was applied to correct the A→T transversion at nucleotide 20 in exon 1 of the β-globin-encoding *HBB* gene in hiPSCs obtained from SCD patients [143]. In these experiments, the positive-negative G418/GCV selection resulted in an average of 85% of colonies with *neo*^R^ targeted insertions with an average of 81% of these colonies presenting the desired *HBB* gene correction [143]. 

The previously described gene editing experiments targeting *LMNA* [142] and *HBB* [143], demonstrated that vector DNA-derived SNPs could be found in the correctly targeted clones at positions 4.4-kb and 3.6-kb away from the *neo*^R^ insertion site within *LMNA* and *HBB* alleles, respectively. On the basis of these results, the authors postulated that the HC-AdV platform might be valuable for repairing mutations found in a relatively broad target region, increasing its potential as a versatile gene correction tool. As an example, a single *LMNA*-targeting HC-AdV could potentially repair over 200 *LMNA* mutations associated with laminopathies [142]. 

Two subsequent studies sought to formally investigate; (i) the extent of homology between endogenous target and exogenous HC-AdV donor templates required for efficient gene editing [144]; and (ii) the relationship between the distance from the knock-in target site and the incorporation of polymorphic markers located along the region of homology [145]. In both studies, HC-AdV targeting constructs were directed to the *CFTR* locus in a hiPSC line harboring the heterozygous mutations ∆F508 and ∆I507 in exon 10 of the target gene. To investigate the effect of the extent of homology on the efficiency of HC-AdV-based gene editing, a set of five different HC-AdVs containing differently sized wild-type *CTFR* sequences were tested [144]. The homology regions spanned total lengths of 23.8 kb, 21.4 kb, 14.8 kb, 9.6 kb, and 5.6 kb. Transduction of hiPSCs with the various HC-AdV donors followed by G418 and GCV double selection led to the emergence of colonies that were subsequently subjected to Southern blot analysis for determining the frequencies of targeted events. The HC-AdV donor construct carrying 23.8 kb of sequence homology to genomic DNA led to 97.4–100% of gene-targeted clones; whilst the HC-AdV donor construct bearing 5.6 kb of sequence homology to genomic DNA yielded 50% of gene-targeted clones [144]. Together, these data lend additional support to a direct correlation between the length of homology between target and donor DNA and the frequency of HDR-mediated gene targeting [21]. 

In order to investigate the extent of exchange of homologous sequences between target and donor DNA templates, twelve 2-bp insertions were introduced along the 23.8 kb homology region in a *CTFR*-targeting HC-AdV construct [145]. Upon HC-AdV-mediated gene targeting, each of these 2-bp insertions convert an endogenous restriction enzyme recognition site into that of another allowing for straightforward assessment of the extent of recombination between target and donor DNA sequences. As assessed through Southern blot analysis, 89.5% of drug-resistant hiPSC clones were correctly targeted at *CFTR* alleles [145]. Furthermore, PCR and restriction enzyme fragment length analyses of the drug-selected hiPSC clones showed that the closest marker to the insertion site (i.e., 208 bp) was incorporated in 100% of the analyzed clones. Conversely, the most distant marker to the insertion site (i.e., 11.2 kb) was incorporated in only 21.7% of the analyzed clones, suggesting that the vicinity of polymorphic markers to the insertion site is proportional to their genomic incorporation rate. Interestingly, 4.8% of the clones presented all the twelve restriction enzyme markers. This data suggests that HC-AdV-based gene editing can be used to introduce genetic information distributed over a wide range of homologous DNA in hiPSCs (i.e., at least up to 22.2 kb) [145].

As aforementioned, HC-AdV-based gene editing is equally applicable for establishing tractable in vitro disease models comprising pairs of isogenic hPSC lines whose genomes differ at well-defined locations (Figure 4). Indeed, HC-AdV-based gene editing has been explored for modeling various human disorders, including; Parkinson’s disease [146], Fanconi anemia [147], retinitis pigmentosa [148], and Werner syndrome [149].

Combining HC-AdV and programmable nuclease technologies offers the prospect for improving gene editing frequencies. In this regard, Suzuki and colleagues used HC-AdVs to deliver donor templates alone or together with TALEN expression units [150]. The TALEN and donor HDR substrates were tailored for targeting *HBB* alleles underlying SCD in hiPSC lines. Transduction of SCD patient-derived hiPSCs with the “all-in-one” HC-AdV resulted in an increase in gene-targeting frequencies when compared to those achieved by HC-AdV delivery of donor DNA templates alone [150]. Specifically, among 2 × 10^5^ cells transduced with the “all-in-one” HC-AdV, 28 G418-resistant clones were analyzed and of these 86% were correctly targeted. Conversely, among 9 × 10^6^ cells transduced with an HC-AdV delivering exclusively donor DNA, 134 G418-resistant clones were analyzed with only 22% of these being correctly targeted [150].

The cumulative data on HC-AdV-based gene editing in hPSCs bodes well for its application in basic research, drug screening, disease modeling, and eventually, development of autologous cell therapies for inherited disorders (Figure 4).

## 4. Conclusions and Outlook

Rapid advancements in the gene editing and stem cell fields are contributing to broaden the range of options for addressing scientific questions and developing candidate gene and cell therapies. To support the integration of these fields, and hence further widen their reach, it is crucial to develop delivery systems that permit introducing programmable DNA-targeting enzymes and donor nucleic acid templates into target cells in an efficient and versatile manner. Moreover, additional parameters that need to be taken into consideration concern the effects that the delivery systems themselves have on the ultimate performance and accuracy of gene editing procedures. In the case of gene-editing tool delivery through viral vector systems, it is important that vector genomes transporting donor templates or encoding programmable DNA-targeting enzymes are refractory to (i) structural rearrangements [103], (ii) epigenetic silencing mechanisms [106,107], and (iii) capture at chromosomal DSBs via illegitimate recombination processes [117,151,152].

Recent developments on genomic engineering comprise the progression from chromosomal cutting to chromosomal non-cutting approaches based on nicking Cas9 variants and on these variants fused to heterologous DNA-modifying moieties. These new gene editing principles include; (i) HDR-mediated chromosomal insertion of exogenous DNA spanning from single bps to whole transgenes through SSB formation at target and donor DNA [61,128,129,130], and (ii) donor DNA-free in situ installation of genetic changes through base editing [131] or prime editing [132]. Base editors, comprising a Cas9 nickase covalently linked to a cytidine or adenine deaminase, induce C→T or A→G transitions, respectively [153,154]. These conversions occur within so-called “editing windows” located in target sequences defined by a standard gRNA [131,153,154]. Prime editors, consisting of a Cas9 nickase covalently linked to an engineered oncoretroviral reverse transcriptase (RT), in addition to transitions, also generate defined indels and transversions, e.g., A→C, G→T, T→A, and C→G [132]. The exact genetic modification depends on the designing of an extended gRNA dubbed prime editor gRNA (pegRNA). The pegRNA is formed by the standard gRNA sequences crRNA and tracrRNA (Figure 1C) covalently linked to a RT primer binding site (PBS) and a RT template sequence bearing the intended edit. After nicking, the PBS locally anneals to the 3′-ended DNA flap that primes RT synthesis over the RT template. The resulting DNA copy of the edit ultimately becomes incorporated at the genomic target site upon a series of cellular processing steps responsible for removing DNA flaps that do not hybridize to target sequences [132].

The SSB-mediated gene editing approaches are opening the perspective for modifying complex genomes with unprecedented precision while minimizing unwanted events characteristic of DSB-mediated gene editing procedures. In addition to off-target mutagenesis [56,57,58,59,60,61], unwanted genome-modifying events include translocations [60,61] and unpredictable genomic “scars” at target sequences in the form of indels and larger structural rearrangements resulting from site-specific DSB repair via prevalent NHEJ pathways [60,155]. Not surprisingly, however, new gene editing approaches and technologies bring to the fore their own sets of shortcomings that need to be carefully assessed and resolved. For instance, base editors can yield off-target editing at the genome and transcriptome levels [156]; whereas primer editing can install target-site mutations derived from RT synthesis into the pegRNA scaffold [132]. Although the optimization of gene editing tools and strategies should ideally take place in the target cell types of interest, each of which bearing its specific epigenome, these investigations are rendered difficult due to the fact that latest-generation gene editing tools are becoming even larger than the original Cas9:gRNA complexes. Indeed, prime editors and base editors consist of a bulky Cas9 nickase fused to one and two, respectively, heterologous proteins that must work together as large macromolecular machines [67,131,132,153,154]. Therefore, there is a pressing need for developing and testing delivery vehicles that can introduce such large machines into primary human cells so that their performance and interaction with human (epi)genomes can be thoroughly investigated. In this context, the research reviewed herein on the testing and use of AdV systems for the targeted genetic modification of stem cells, progenitor cells, and their progeny, supports the view that these agents will become increasingly applied for achieving flexible gene-editing tool delivery and precise gene-editing outcomes in human cells. Defining features underpinning the suitability of AdVs for investigating new gene-editing modalities include their efficient transduction of cycling and quiescent cells, amenability to tropism modifications, high genetic stability and strict episomal nature. Moreover, in the case of HC-AdVs, the absence of viral genes and vast packaging capacity (i.e., up to 36 kb) makes this platform particularly suited for ferrying into cells large genetic payloads for testing precision gene-editing principles based on the recruitment of the HDR pathway or the delivery of DNA-editing fusion constructs, e.g., base and prime editors.
